# Non-bacterial cystitis following treatment with toripalimab for alpha-fetoprotein-producing gastric adenocarcinoma: a case report

**DOI:** 10.3389/fimmu.2026.1836979

**Published:** 2026-07-14

**Authors:** Zhenpeng Li, Xiuxiu Yi, Jie Fu, Yan Liang, Sensen Zhang, Xv Yang, Zhonghai Du

**Affiliations:** 1 First Clinical Medical College of Shandong University of Traditional Chinese Medicine, Jinan, Shandong, China; 2 Weifang Municipal Hospital of Traditional Chinese Medicine, Weifang, Shandong, China

**Keywords:** alpha-fetoprotein-producing gastric carcinoma, cystitis, immune checkpoint inhibitors, immune-related adverse events, toripalimab

## Abstract

Immune checkpoint inhibitors (ICIs) have revolutionized the management of gastric cancer; however, they can lead to rare immune-related adverse events (irAEs) affecting the urinary system. Herein, we report a case of non-bacterial cystitis complicated by acute kidney injury (AKI) in a 59-year-old male patient with Alpha-fetoprotein-producing gastric carcinoma (AFP-GC). Following treatment with toripalimab combined with SOX chemotherapy, the patient developed urinary tract irritation symptoms, gross hematuria, and stage II AKI. Cystoscopy revealed diffuse mucosal hemorrhage, and biopsy demonstrated extensive infiltration of CD3^+^, CD8^+^, CD4^+^, and CD20^+^ lymphocytes, along with high PD-L1 expression and TIA-1 positivity, confirming the diagnosis of non-bacterial cystitis. A full-dose methylprednisolone pulse of 200 mg/day effectively alleviated the symptoms and restored renal function. This case underscores the importance of vigilance for urinary system irAEs in patients receiving ICIs, emphasizing that early identification and systematic evaluation are critical. Full-dose corticosteroids should be used for moderate to severe irAEs. Moreover, this report provides valuable insights into immunotherapy practice and toxicity management in the rare AFP-GC subtype.

## 1 Introduction

Immune checkpoint inhibitors (ICIs) that target programmed death receptor-1 (PD-1) have significantly enhanced the prognosis of advanced gastric cancer (1). Nonetheless, by disinhibiting T-cell activity, ICIs may provoke immune-related adverse events (irAEs) in nearly every organ (2), with the incidence significantly escalating when used in conjunction with chemotherapy (3). Although irAEs impacting the skin, gastrointestinal tract, and endocrine system are well-documented, involvement of the urinary system—especially non-bacterial cystitis—remains comparatively rare, and there is a lack of consensus concerning its diagnosis and management. Alpha-fetoprotein-producing gastric carcinoma (AFP-GC) is an uncommon and extremely aggressive subtype characterized by hepatoid differentiation, comprising between 1.5% to 15% of all gastric malignancies. It is marked by early metastases and unfavorable prognosis (4–6). The ideal therapy for AFP-GC is yet to be established, and experience with ICIs in this subtype is scarce. Moreover, the scope of immune-related adverse events in patients with AFP-GC has not been extensively delineated. This report details a case of locally advanced AFP-GC in which the patient experienced uncommon non-bacterial cystitis and subsequent acute kidney injury (AKI) following neoadjuvant treatment with toripalimab and SOX chemotherapy. This instance is unique in multiple respects. Initially, It primarily focuses on AFP-GC, a rare tumor subtype, hence enhancing the little clinical data about immunotherapy in this population. Secondly, the manifestation of non-bacterial cystitis, an uncommon urinary immune-related adverse event that concurrently affected the upper urinary tract and resulted in acute kidney injury, offers a comprehensive clinical-pathological evidence chain for this toxicity. This paper seeks to provide evidence-based recommendations for professionals overseeing immunotherapy and related complicated adverse events in AFP-GC.

## 2 Case report

**Figure 1:**
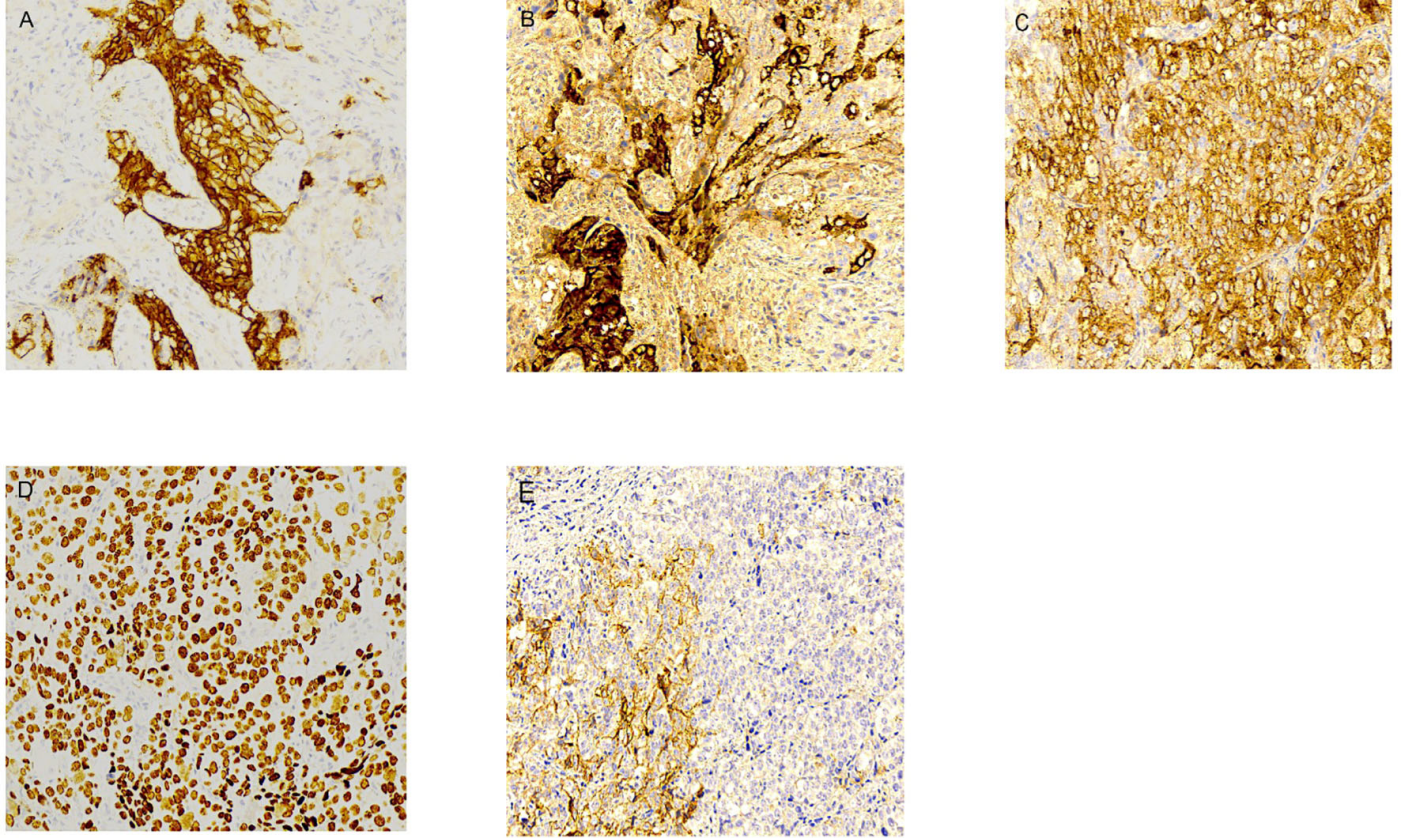
Immunohistochemistry of a gastric adenocarcinoma producing alpha-fetoprotein. Magnification is 200. **(A)** HER2 (3+) **(B)** AFP (+) **(C)** GPC-3 (+) **(D)** SALL-4 (+) **(E)** Claudin 18.2 (20%+).

**Figure 2:**
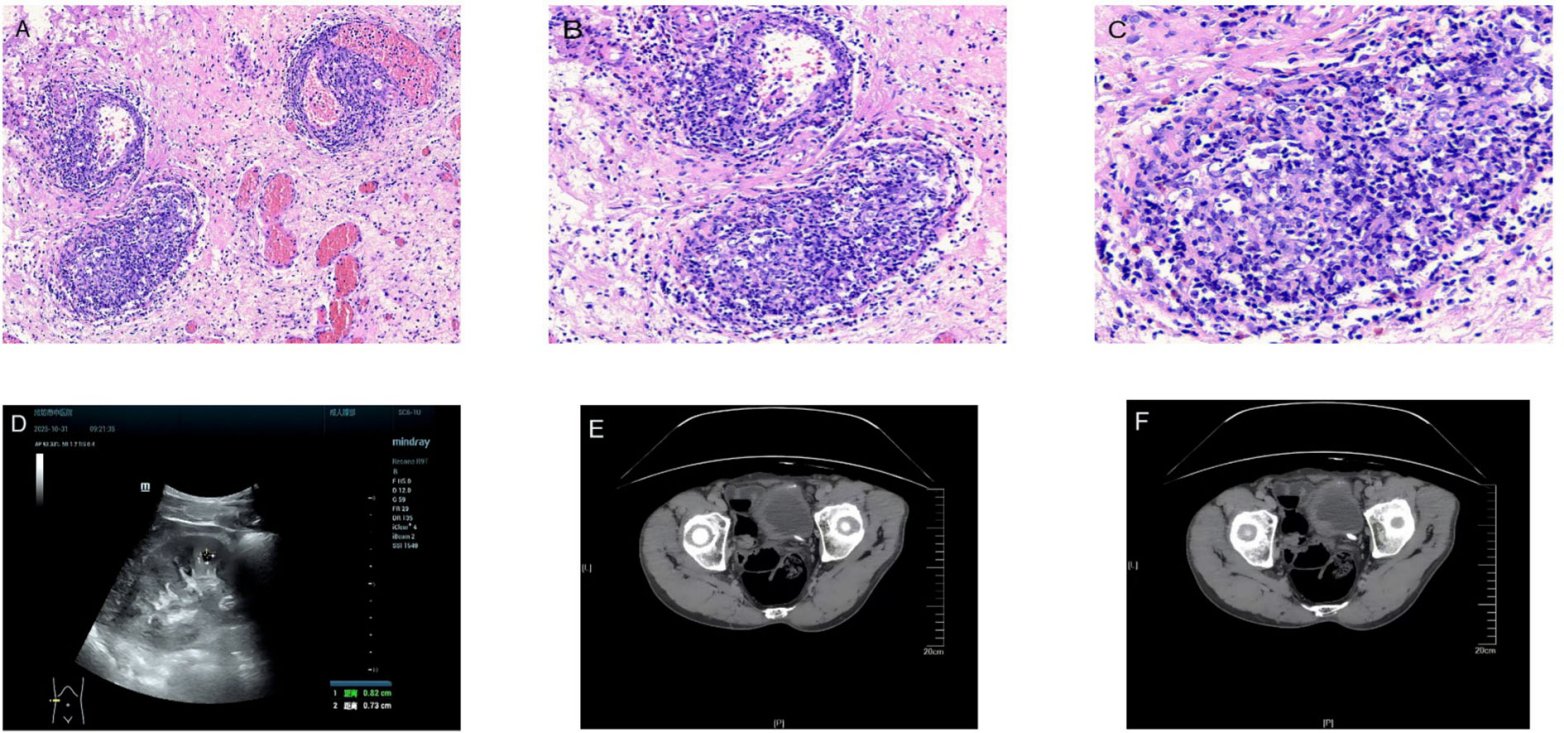
Pathological analysis following radical gastrectomy. Hematoxylin and eosin staining. **(A)** Magnification is 100, **(B)** Magnification is 200, **(C)** Magnification is 400.Imaging findings of the urinary system: **(D)** Mild dilation of the bladder and ureters on ultrasound; **(E, F)** CT scan showing thickening of the bladder wall.

**Figure 3:**
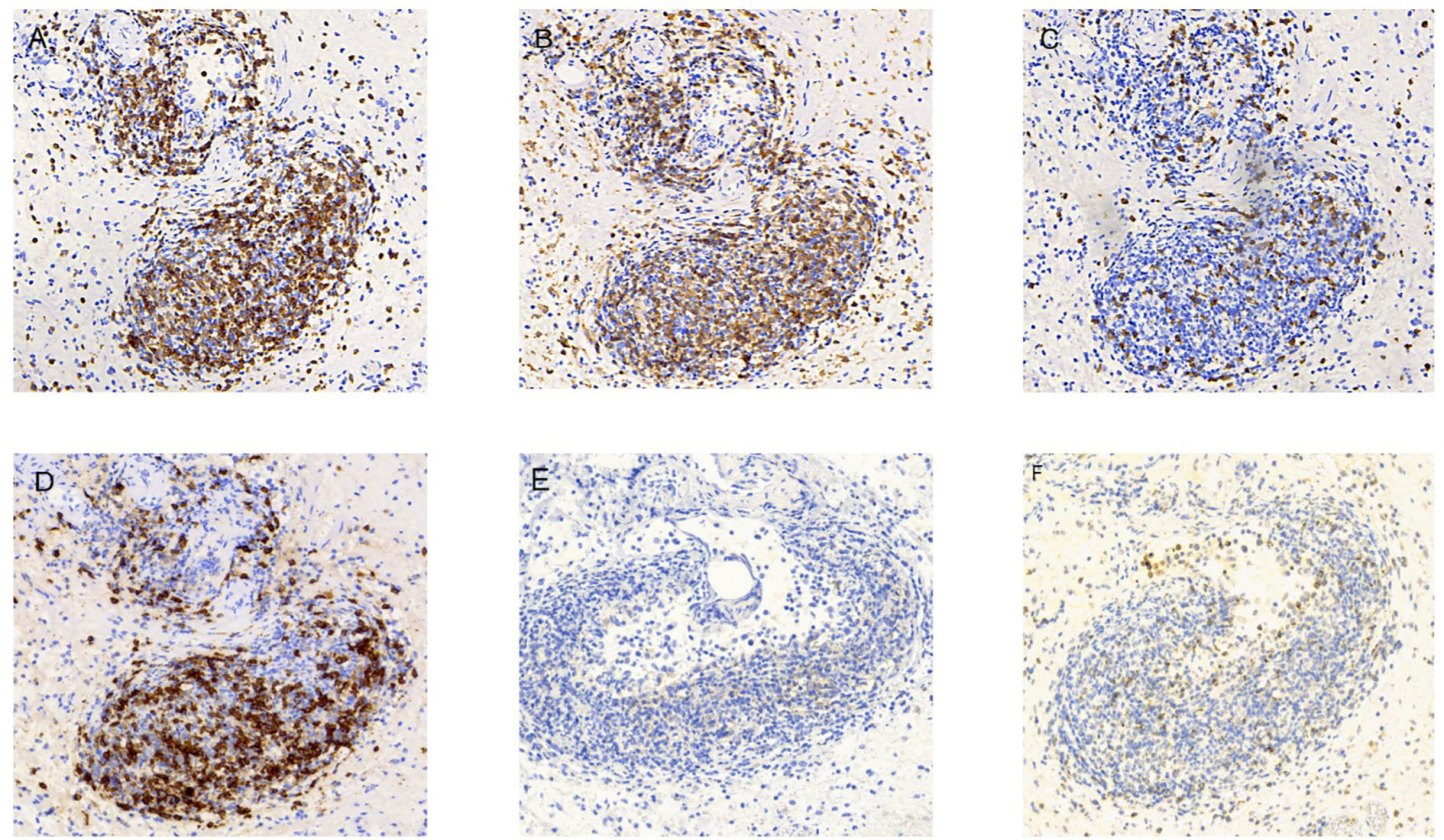
Biopsy of bladder occupation. **(A)** T lymphocytes, positive staining for CD3. **(B)** T lymphocytes, positive staining for CD4. **(C)** T lymphocytes, positive staining for CD8. **(D)** B lymphocytes, positive staining for CD20. **(E)** T lymphocytes, positive staining for PD-L1. **(F)** T lymphocytes, positive staining for TIA-1.

**Figure 4:**
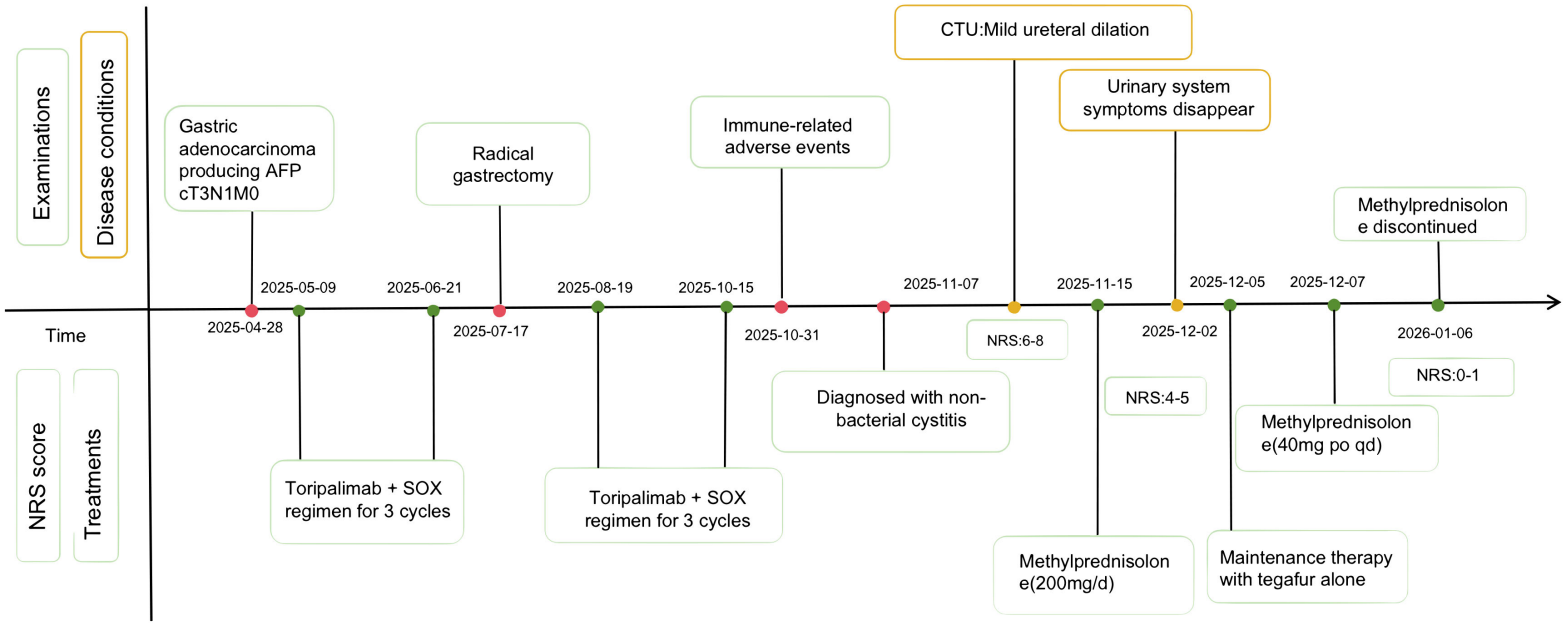
The timeline of the patient’s symptom, diagnosis and treatments.

A 59-year-old male reported in April 2025 with nocturnal epigastric discomfort. The physical examination indicated an ECOG performance status of 1, stable vital signs, and the absence of palpable abdominal tumors or lymphadenopathy. The patient’s partner has lung cancer. He possesses no history of food or drug allergies, nor has he ever engaged in smoking or alcohol consumption. He is not afflicted with diabetes, hypertension, renal illness, or hepatitis, nor does he have a history of autoimmune disorders; additionally, there is no familial history of cancer. A gastroscopy conducted on 28 April 2025 identified a lump in the stomach’s antrum. The pathological analysis of the biopsy samples verified poorly differentiated adenocarcinoma. Immunohistochemistry revealed HER2 (3+) expression and preserved mismatch repair proteins (pMMR). The tumor cells exhibited positivity for AFP, GPC3, SALL4, and Claudin18.2, with moderate-to-strong positivity observed in roughly 20% of the tumor cells (**Figure 1**). Serum alpha-fetoprotein was significantly high at 532.6 μg/L (normal <12 μg/L), confirming the diagnosis of AFP-GC. CT and PET/CT demonstrated thickening of the stomach antral wall accompanied by heightened metabolic activity and several enlarged lymph nodes in the hepatogastric region, indicative of clinical stage cT3N1M0 (Stage III).The patient underwent three cycles of neoadjuvant therapy utilizing toripalimab (240 mg, day 0) in conjunction with the SOX regimen (oxaliplatin 220 mg on day 1 and tegafur 60 mg administered twice daily from day 1 to day 4) from 9 May to 21 June 2025.Post-neoadjuvant therapy restaging CT revealed SD. The patient later underwent laparoscopic-assisted radical gastrectomy on July 17, 2025. Postoperative pathology indicated the absence of remaining active cancer cells in the stomach mucosa, resection margins, or any of the assessed lymph nodes (0/21), resulting in a pathological complete response (pCR) (**Figures 2A–C**). After three cycles of adjuvant therapy with the identical regimen (August 19 to October 15, 2025), the patient had significant immune-related side effects. Clinical symptoms comprised urine frequency, urgency, dysuria, significant hematuria with clots, and dull kidney discomfort. The physical examination indicated soreness in the bilateral costovertebral angles. Numerical Rating Scale (NRS) score of 6–8. Laboratory assessment revealed pyuria (urine white blood cells 2+), hematuria (red blood cells 2+), and proteinuria (3+), with negative urine cultures suggesting sterile inflammation. The patient had oliguria (urine output <0.5 mL/kg/h for >12 hours) and increased serum creatinine (144 μmol/L), fulfilling the criteria for stage 2 acute kidney injury (AKI).Cystoscopy and imaging demonstrated (CTU) widespread mucosal hyperemia and bleeding in the bladder, accompanied by bilateral ureteral wall thickening and dilation(**Figures 2D–F**). The bladder biopsy revealed significant infiltration of CD3^+^, CD4^+^, CD8^+^, and CD20^+^ cells along the mucosal-submucosal interface, together with elevated PD-L1 expression and TIA-1 positivity (**Figure 3**). The patient was diagnosed with immune checkpoint inhibitor (toripalimab)-associated cystitis complicated by acute kidney injury, based on clinical presentation, imaging findings, cystoscopic appearance, and pathology data. On 31 October 2025, the patient underwent extensive laboratory examinations, which clinically excluded non-immune cystitis. On November 4th, the patient was referred to the Department of Urology for a cystoscopy and pathological biopsies.(**Figures 2D–F**, **3**). Commencing November 15, methylprednisolone 200 mg/day (100 mg every 12 hours) was delivered as enhanced pulse treatment. Six days later (21 November), symptoms began to ameliorate, allowing for a gradual reduction: 100 mg/day for 5 days, 80 mg/day for 5 days, and 60 mg/day for 5 days. Oral methylprednisolone was then maintained at 40 mg/day with a gentle tapering schedule, decreasing by 4 mg every 3 days. By January 6, 2026, all urinary symptoms had abated, and steroid therapy was terminated. No further episodes of similar pain or urinary symptoms occurred during follow-up. The patient’s pathology achieved pCR, and since December 3, 2025, he have been receiving single-agent Tegafur (60 mg bid d1-d4), with the regimen continued as ongoing treatment. The timeline of treatment course was summarized in **Figure 4**.

## 3 Discussion

Non-bacterial cystitis is an uncommon irAE. The vague clinical presentation, resembling bacterial cystitis and upper urinary tract infection, complicates early differential diagnosis significantly (7). The diagnosis in this case was determined based on the established criteria for immune-related cystitis, which included: a documented history of ICI exposure; consistently confirmed sterile pyuria; characteristic imaging and cystoscopic findings, such as diffuse bladder mucosal hyperemia and hemorrhage, as well as bilateral ureteral wall thickening and dilation; and, importantly, histopathological evidence revealing extensive inflammatory infiltration of the bladder mucosal stroma, primarily consisting of CD3^+^, CD4^+^, CD8^+^, and CD20^+^ T lymphocytes, alongside elevated PD-L1 expression (7–10). Alternative etiologies, such as infectious, calculous, and metastatic origins, were methodically ruled out. The patient had significant clinical improvement after the cessation of immune checkpoint inhibitors and the commencement of glucocorticoid therapy (8, 11). The detection of TIA-1 positive signified cytotoxic T-cell activity, further corroborating an immune-mediated pathophysiology (12).

The primary cause of irAEs entails abnormal activation of the immune system, resulting in unintended damage to normal tissues, but the exact molecular pathways have yet to be completely clarified (13). Concerning the etiology of non-bacterial cystitis, current evidence indicates a misdirected activation of T-cells targeting antigens co-expressed by both tumor cells and normal urothelial cells (8, 14). In our patient, PD-L1 expression was detected on urothelial cells, a result that aligns with other studies and may elucidate the urinary tract’s vulnerability to immune-mediated damage (15). The significant presence of CD3^+^ and CD8^+^ T cells in bladder biopsy samples, along with TIA-1 positivity, robustly indicates a cytotoxic T-cell-mediated mechanism (12).Significantly, despite the lack of tumor cell infiltration observed in both imaging and histological assessments, urothelial cells demonstrated elevated PD-L1 expression, indicating that the urothelium may function as a direct target of immune assault (14). This behavior can be ascribed to interferon-gamma (IFN-γ) generated by many invading CD3^+^ and CD8^+^ lymphocytes, recognized for their significant cytotoxic effects on urothelial cells (11). The diffuse thickening of the ureteral wall and the resultant AKI in this case likely stemmed from ascending inflammation in the urinary system or functional impairment due to this condition. These findings highlight the necessity of a systematic assessment of the upper urinary tract when immune-mediated cystitis is suspected (10).

Since its initial description in 1970, the distinctive biological properties of AFP-GC have been gradually clarified (4–6). The etiology of this unique entity can be ascribed to the common embryonic foregut origin of the stomach and liver, where gastric cancer cells inappropriately activate hepatocyte-related genes during dedifferentiation (16, 17). Immunohistochemical profiling often demonstrates positive for AFP, GPC3, and SALL4, with most patients exhibiting significantly high serum AFP levels. Prior research has shown that increased serum AFP is substantially associated with the quantity of CD4^+^ T lymphocytes, neutrophils, and inflammatory cytokines. The tumor immune microenvironment of AFP-GC is notably marked by diminished immunological activity, perhaps fostering a pro-tumorigenic environment (18). Changes in the primary tumor microenvironment can facilitate distant tumor spread and colonization by triggering systemic immune-inflammatory responses (19). While distant metastases were not detected in this case, several lymph node metastases were apparent in the hepatogastric region. The potential role of this changed systemic immune state in creating an immunological propensity for the formation of irAEs is a compelling subject that requires additional exploration.

High-dose glucocorticoids are fundamental in the management of mild to severe irAEs (20). In this case, the situation was promptly managed with a full-dose steroid shock, followed by durable remission with moderate, stepwise reduction. This indicates that in some severe or resistant instances, prompt and more assertive modification of immunosuppressive methods may be necessary, and the incorporation of second-line immunomodulatory drugs should be contemplated. This occurrence was characterized as a grade 3 adverse reaction per the Common Terminology Criteria for Adverse Events (CTCAE) version 6.0, requiring temporary cessation of ICI medication, in accordance with current management guidelines (21, 22). Epidemiological findings indicate that male sex and a history of autoimmune disease may correlate with an elevated risk of irAEs, providing essential insights for therapeutic risk stratification (23, 24).

This report possesses multiple limitations. The findings are not generalizable due to the nature of this single case investigation. The lack of renal biopsy hindered a conclusive evaluation of concurrent interstitial nephritis; nevertheless, the swift reaction to corticosteroid treatment and the amelioration of acute kidney injury imply that the diseased process was primarily confined to the urinary tract. Additionally, extended follow-up beyond six months is essential to assess potential late recurrence or sequelae.

## 4 Conclusion

This case report details an uncommon instance of AFP-GC exacerbated by non-bacterial cystitis and acute renal injury subsequent to treatment with toripalimab in conjunction with chemotherapy. The example highlights the necessity for increased awareness regarding uncommon immune-related side effects when urine symptoms arise in patients undergoing immunotherapy. Diagnosis relies on cystoscopic biopsy, which generally demonstrates distinctive lymphocytic infiltrate and elevated PD-L1 expression. Full-dose corticosteroids should be used for moderate to severe irAEs. This research enhances the sparse literature on immune-related adverse events impacting the urinary tract and offers pragmatic recommendations for their diagnosis and therapy.

## Data Availability

The original contributions presented in the study are included in the article/**Supplementary Material**. Further inquiries can be directed to the corresponding author.
